# A Survey of Visible-Light-Communication-Based Indoor Positioning Systems

**DOI:** 10.3390/s24165197

**Published:** 2024-08-11

**Authors:** Ruofan Wang, Guanchong Niu, Qi Cao, Chung Shue Chen, Siu-Wai Ho

**Affiliations:** 1 Guangzhou Institute, Xidian University, Guangzhou 510530, China; 23011211049@stu.xidian.edu.cn (R.W.); niuguanchong@xidian.edu.cn (G.N.); caoqi@xidian.edu.cn (Q.C.); 2 Nokia Bell Labs, Paris-Saclay Center, 91300 Massy, France; 3 Teletraffic Research Centre, University of Adelaide, Adelaide, SA 5005, Australia; siuwai.ho@adelaide.edu.au

**Keywords:** visible light communication, indoor positioning system, localization scheme, system design, performance comparison

## Abstract

There is a growing demand for indoor positioning systems (IPSs) in a wide range of applications. However, traditional solutions such as GPS face many technical challenges. In recent years, a promising alternative has been emerging, the visible light communication (VLC)-based IPS, which offers a combination of high accuracy, low cost, and energy efficiency. This article presents a comprehensive review of VLC-based IPSs, providing a tutorial-like overview of the system. It begins by comparing various positioning systems and providing background information on their inherent limitations. Experimental results have demonstrated that VLC-based systems can achieve localization accuracy to within 10 cm in controlled environments. The mechanisms of VLC-based IPSs are then discussed, including a comprehensive examination of their performance metrics and underlying assumptions. The complexity, operating range, and efficiency of VLC-based IPSs are examined by analyzing factors such as channel modeling, signal processing, and localization algorithms. To optimize VLC-based IPSs, various strategies are explored, including the design of efficient modulation schemes, the development of advanced encoding and decoding algorithms, the implementation of adaptive power control, and the application of state-of-the-art localization algorithms. In addition, system parameters are carefully examined. These include LED placement, receiver sensitivity, and transmit power. Their impact on energy efficiency and localization accuracy is highlighted. Altogether, this paper serves as a comprehensive guide to VLC IPSs, providing in-depth insights into their vast potential and the challenges that they present.

## 1. Introduction

Mobile devices have become essential tools for communication in our daily lives, and the utilization of positioning systems is becoming increasingly common. These systems facilitate the precise localization of mobile devices, thereby supporting a range of applications, including tracking [1], navigation [2], social networking [3], and augmented reality [4]. In addition, positioning systems also play a crucial role in ubiquitous computing [5], as well as emergency services [6]. In general, positioning systems can be classified into two categories: indoor and outdoor systems. In outdoor scenarios, the Global Positioning System (GPS) [7] is the most widely used and well-developed technology. It leverages a worldwide satellite network to offer precise user location information and turn-by-turn navigation. However, in indoor environments, the various obstacles, like roofs, walls, and equipment, significantly weaken and scatter satellite signals. Additionally, the effects of multipath interference and other sources of interference can lead to positioning errors of several meters [8]. Despite efforts to enhance accuracy through the introduction of high-sensitivity GPSs, achieving satisfactory results in indoor settings remains a challenge [9].

Undoubtedly, a wide range of applications significantly benefit from indoor location services. In our daily lives, the presence of large buildings with complex interiors, such as hospitals, airports, supermarkets, university campuses, and the like, often poses a formidable challenge to visitors who invest a great deal of time and effort in navigating them. An indoor positioning system (IPS) emerges as a vital ally in such circumstances, affording invaluable assistance and introducing an added layer of value. A typical example is the application for museums, where an IPS utilizes the visitor’s position to offer advice and guidance, enabling them to immerse themselves in the exhibits and enhance their overall experience [10].

In addition to the indispensable role of an IPS in navigation, it also serves as a desirable tool for tracking purposes, particularly for monitoring the whereabouts of portable mobile devices, such as wheelchairs in hospitals. This duality of functionality provides multiple benefits. On the one hand, patients can promptly access enhanced services exactly when needed, fostering a sense of immediacy and efficacy. On the other hand, the IPS effectively enhances the traceability of expensive devices, improving their security and reducing the risk of loss. It is worth noting that the availability of high-precision positioning information is of paramount importance in areas such as robotics and the Internet of Things (IoT) [11,12], in accordance with the extensive references provided therein.

Because of the growing potential and evolving applications of indoor positioning, there has been extensive discussion in recent years. An exemplary initiative in this area is the In-Location Alliance (ILA) [13], an organization founded by leading companies in the mobile communications industry. Its primary goal is to drive innovation and cultivate cutting-edge indoor positioning services characterized by exceptional accuracy and minimal power consumption, even when users move.

Notable companies such as Google, Apple, and Microsoft [14] have embarked on the development of novel indoor positioning solutions. This has further fueled the discourse in this area. Numerous technologies have been the subject of intense discussion, including VLC, infrared (IR), radio frequency identification (RFID), ultrasound, wireless local area network (WLAN), Bluetooth, sensor networks, ultra-wideband communication (UWB), magnetic signals, and others. In Section 3, we will delve into a comprehensive exploration of these technologies.

In this article, we focus on VLC-based IPSs. Our primary goal is to summarize the latest breakthroughs and significant findings in this emerging field of research. The overall organization of this paper is summarized as follows. In Section 2, the various applications of VLC-based IPSs are highlighted. Section 3 introduces the basic principles of VLC-based IPSs, while drawing insightful comparisons with alternative positioning methods. Section 4 presents an in-depth exploration of the latest advances in VLC-based positioning techniques. In addition, the overarching algorithms will be succinctly encapsulated in Section 5, which effectively classifies the multitude of recent research papers into six distinct algorithmic paradigms. Furthermore, we do not want to neglect the challenges that researchers face in this area. Thus, Section 6 serves as a comprehensive repository that both discusses these obstacles and provides recommendations for future endeavors. This paper is concluded in Section 7.

## 2. Applications for VLC-Based IPS

**Figure 1:**
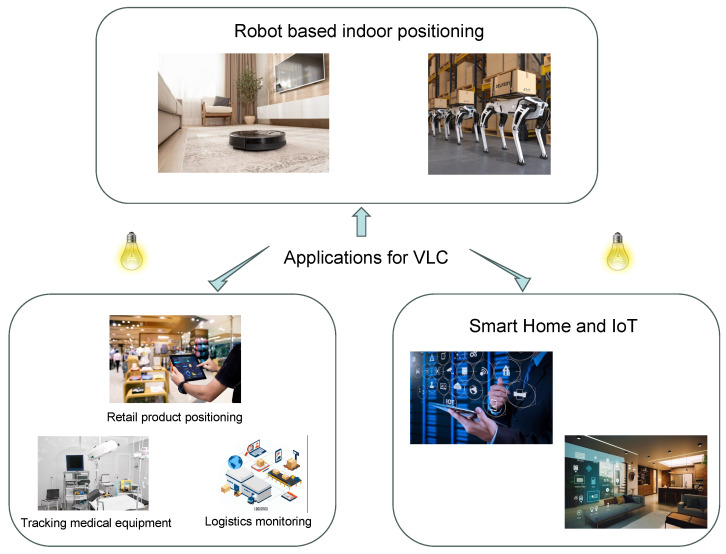
Applications of VLC-based IPS.

VLC-based IPSs have a wide range of applications, as shown in Figure 1. In retail, they can be used to enhance the customer experience by adding location-specific amenities and customized suggestions. In healthcare, VLC-based IPSs can be used to facilitate patient tracking and monitoring, thereby enhancing effectiveness and ensuring well-being. In industrial domains, these systems act as catalysts, enabling seamless asset monitoring and streamlining workflow management. In addition, VLC-based IPSs can be used in transportation networks to provide real-time passenger monitoring and seamless navigation. Taken together, these pioneering systems provide unprecedented indoor positioning accuracy and adaptability, enabling industries to optimize operational efficiency, enhance security, and deliver customized services.

### 2.1. Indoor Navigation

As one of the most important applications, VLC-based indoor navigation utilizes VLC-enabled lighting fixtures to provide location-based information and navigation within indoor spaces. By leveraging existing lighting equipment, VLC facilitates accurate positioning and navigation for users. In [15,16,17,18,19], the robots are designed to perform indoor navigation using VLC-based methods.

The indoor navigation system based on VLC works by installing VLC transmitters in the form of LED lights throughout a building or indoor space [18]. These transmitters emit light signals that contain unique identifiers or location information. Robots can then receive these signals on their mobile devices equipped with VLC receivers, such as cameras or light sensors [17]. To navigate indoors using VLC, robots simply need to open a compatible mobile application or use a device that supports VLC reception. The applications will detect the VLC signals from the surrounding lights and determine the user’s location based on the received signals. The position of robots can be displayed on a map or through augmented reality overlays, providing real-time guidance and directions.

VLC-based indoor navigation offers several advantages [20]. First, it does not require additional infrastructure because it uses existing lighting. Second, VLC signals do not interfere with other wireless technologies such as Wi-Fi or Bluetooth, making them suitable for use in environments with high radio frequency interference. In addition, VLC can provide accurate positioning even in areas where GPS signals are weak or unavailable, such as homes and markets.

### 2.2. Asset Tracking

Asset tracking uses LED lights to transmit signals for indoor asset positioning and tracking. Asset tracking for VLC offers several advantages over traditional tracking methods [21]. Similar to scenarios of indoor navigation, it implements the existing LED lighting infrastructure, reducing the need for additional hardware investment. This makes it a cost-effective solution for asset tracking in various industries, including warehousing [22], logistics [23], retail [24], healthcare [25], and manufacturing [26].

One of the key benefits of VLC-based asset tracking is its high accuracy. By using multiple LED lights strategically placed throughout the indoor environment, the position of assets can be determined with great precision. This enables the real-time tracking and monitoring of assets, improving inventory management and reducing the risk of loss or theft. In hospitals, VLP systems can be used to track patients and equipment to ensure the safety and traceability of medical equipment. We carried out tests in a medical laboratory and achieved the positioning and monitoring of medical equipment with a VLP system with a positioning error of less than 1 cm. Another advantage of VLC-based asset tracking is its low interference. VLC operates in the visible light spectrum, which is not crowded with other wireless signals such as Wi-Fi or Bluetooth. This ensures reliable and interference-free communication, resulting in more accurate asset tracking results.

VLC-based asset tracking systems are also highly scalable [27]. Additional LED lights can be easily installed to expand the coverage area or increase the density of tracking points. This flexibility allows companies to tailor the system to their specific needs and easily scale as their operations grow.

### 2.3. Smart Homes and IoT

VLC-based indoor positioning can indeed be integrated into smart home systems to enable location-aware automation. By using visible light signals emitted from LED lights, VLC technology can accurately determine the position of individuals within a house. This information can then be used to trigger various automation actions based on the user’s location [28]. For example, when a user enters a room, the system can automatically adjust the lighting, temperature, and sound settings to provide the best comfort experience.

Specifically, personalized settings can be applied based on the occupant’s location within the house. For example, the temperature, music, or preferred lighting ambiance can be adjusted according to the occupant’s preferences in different areas of the house. Therefore, VLC-based indoor positioning can greatly enhance the overall smart home experience by making automation more context-aware and personalized. It adds an extra layer of intelligence to the system, allowing it to adapt and respond to the user’s location in real time [29].

## 3. Technologies for Indoor Positioning Systems

In this section, we begin with an elegant overview of VLC-based IPSs. After that, we explore alternative positioning methods, carefully examining their advantages and limitations in comparison to VLC-based IPSs. Through this examination, we aim to uncover the distinct advantages and distinguishing characteristics that make VLC-based IPSs a cutting-edge technology in the field of indoor positioning.

### 3.1. What Are VLC-Based IPSs

VLC harnesses the power of visible light, which falls within the wavelength spectrum of 380 nm to 780 nm, to enable seamless data transmission. This transformative technology, which can be traced back to the 1880s [30], has seen remarkable advances in recent decades. To promote its widespread adoption and further development, IEEE 802.15.7 [31] has meticulously defined the physical (PHY) and medium access control (MAC) layer standards for both broadcast and bi-directional communications. VLC is more than just a viable alternative to traditional RF communications, particularly Wi-Fi. With the ongoing transition from fluorescent lighting to energy-efficient light-emitting diodes (LEDs), VLC, often referred to as light fidelity (Li-Fi), holds great promise for revolutionizing indoor wireless connectivity [32]. These LED-based lighting infrastructures not only provide superior illumination but also have the potential to support high-speed data links. It is worth noting that the convergence of LEDs and lighting infrastructure presents exciting opportunities for the development of novel positioning and communication systems [33]. By leveraging the existing LED infrastructure, innovative positioning solutions can be developed, expanding the horizons of indoor localization.

**Figure 2:**
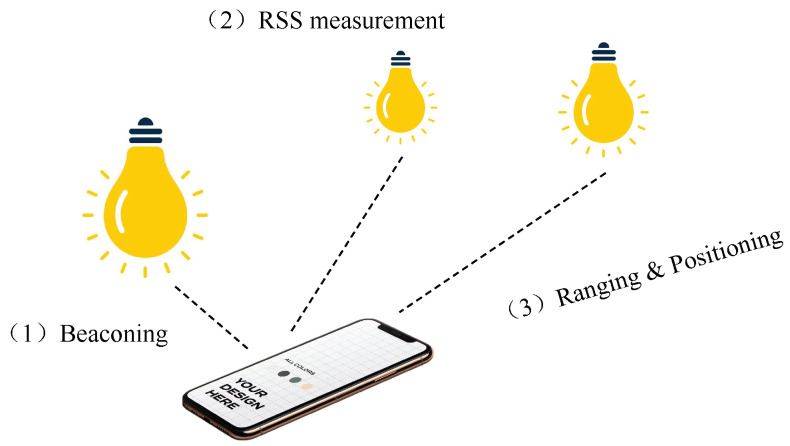
Example of a VLC indoor positioning system: Epsilon [14].

VLC-based IPSs benefit from breakthrough LED technology. For example, this article [34] describes how to improve the performance of visible light communication (VLC) systems by optimizing the superlattice interlayers (SLs) of Si-based LEDs. By receiving information such as the position of transmitters and detecting the direction and intensity of the received light, the position of the receiver can be estimated. The position of transmitters is usually broadcast using VLC. The driving force behind VLC-based IPSs is the use of existing and future LED infrastructure, as well as the integration of state-of-the-art smart mobile devices equipped with light sensors (see, e.g., [35]). By leveraging these resources and developing sophisticated algorithms, novel indoor positioning systems can be created; see Figure 2 in reference [14] for an illustrative example.

In VLC-based IPSs, the primary choice for transmitters revolves around LEDs, as opposed to incandescent or fluorescent lamps/tubes, in line with the future trend of lighting systems. LEDs offer significant advantages, including energy efficiency and a long lifespan [36,37,38]. Furthermore, communications using LEDs enables high data rates due to their ability to support modulation with higher spectral efficiency.

As for receivers, mobile phones have become one of the most prevalent and indispensable user devices in the 21st century [39]. Leveraging the convenience and ubiquity of mobile phones, various design solutions can be explored. For example, the integration of a mobile phone’s imaging camera, photodetectors (PDs), inertial sensors, or new light sensing functions can greatly enhance the receiver capabilities in VLC-based IPSs. Further technical details on these solutions are discussed in Section 4.

### 3.2. Other Methods

Apart from the widely used positioning method named GPS, which is the most discussed in Section 1, there are a variety of alternative solutions [40]. These alternative methods and their performance can be evaluated and analyzed by referring to the relevant literature [40] and corresponding references. When considering positioning systems, it is crucial to take into account various criteria and considerations, such as accuracy, cost, information requirements, computational complexity, and user privacy. For further discussion, readers are encouraged to explore the details presented in [41]. Table 1 presents a comprehensive overview of the major positioning methods, highlighting their accuracy and cost in parallel with VLC-based IPSs. Subsequent discussions will delve into the specifics of each method.

Cellular-based positioning systems are more commonly used outdoors than indoors. Similar to GPS, a mobile user uses signals received from base stations to determine its own position. In addition, base stations can also use signals received from mobile users and combine them at a central coordinator to estimate the mobile’s position [42]. The positioning accuracy in these systems is affected by factors such as the density of base stations and the positioning methods, including received signal strength (RSS), angle of arrival (AOA), time of arrival (TOA), or a combination of these. It is important to note that sources of positioning error include non-line-of-sight (NLOS) propagation, multipath propagation, and multiple access interference. Despite the progress that has been made, there are still several technical challenges that must be overcome to achieve accurate positioning.

Wi-Fi-based positioning systems typically involve adding a location server to an existing WLAN infrastructure. These systems use methods such as RSS or fingerprinting for positioning measurements [43], and further discussion can be found in the references therein. In order to implement Wi-Fi-based positioning systems, centralized entities such as fingerprint databases of Wi-Fi signals are required. Therefore, site measurement of Wi-Fi signals to build the database is inevitable during initial deployment or when relocating access points (APs), but it is labor-intensive and time-consuming. Regarding RFID systems [44,45,46], passive RFID cards are unable to provide detailed high-resolution localization within a room. On the other hand, active RFID cards are more practical for indoor positioning systems because they regularly transmit signals to receivers in a given area, enabling positioning. However, these systems often have slow response times and relatively long latency compared to other methods.

Both infrared (IR)- and Bluetooth-based systems are designed for short-range, low-power communications. As a result, they are not well suited for user mobility and accurate positioning. An exploration of hybrid Wi-Fi and Bluetooth systems for indoor positioning can be found in [47]. UWB- or ultrasound-based systems can provide high positioning accuracy, but their cost is significantly higher than other alternatives. In contrast to other positioning systems, VLC-based systems have several advantages. First, the accuracy of VLC systems meets the requirements of high-resolution indoor localization while keeping costs low because of the existing lighting infrastructure. In addition, VLC systems use the license-free visible light spectrum, eliminating the need for additional licenses. Second, VLC-based systems are environmentally friendly because they use energy-efficient LEDs as transmitters, resulting in low power consumption and minimal radio frequency interference.

## 4. Techniques of VLC-Based Indoor Positioning Systems

This section introduces different techniques of VLC-based IPSs, including RSS, TDOA, AOA, etc. For various indoor scenarios, these methods offer accurate and reliable location estimation with minimal infrastructure modification while providing benefits of increased energy efficiency and security.

### 4.1. Channel Model

This part presents the channel model of the VLC-based channel model for LEDs. A general VLC channel model comprises two primary components, namely the propagation paths and channel characteristics. The paths may include line-of-sight (LOS) and NLOS components, with both direct and reflected transmissions. Channel characteristics include path loss, delay spread, and channel gain, which are influenced by the environment and system design.

#### 4.1.1. Direct Light

To consider the propagation paths of LEDs, our initial focus shall be solely dedicated to the directed light. The channel link gain is given by [48]:

(1) $$\begin{array}{c} G_{d}=\left\{\begin{array}{cc} & \frac{\left(m+1\right)A}{2\pi {D_{d}}^{2}}\cos^{m}\left(\phi \right)T\left(\theta \right)g\left(\theta \right)\cos\left(\theta \right),\;\;\mathrm{if}\;\left|\frac{\theta }{FOV}\right|\leq 1,\\ & \;\;\;\;\;\;\;\;\;0,\;\;\;\;\;\;\;\;\;\;\mathrm{if}\;\left|\frac{\theta }{FOV}\right|>1 \end{array}\right. \end{array}$$

where the parameters are illustrated in Table 2.

The received power is thus given by

(2) $$\begin{array}{c} P_{rd}=G_{d}\cdot P_{t} \end{array}$$

where $P_{rd}$ denotes the received power and $P_{t}$ denotes the transmit power.

#### 4.1.2. Reflected Light

Since the light can be reflected by walls or other objects and then captured by the receiver, we have the aggregate received power given by

(3) $$\begin{array}{c} P_{r}=P_{rd}+P_{rnon-d}=G_{d}\cdot P_{t}+\int_{wall}P_{t}\;dG_{ref} \end{array}$$

where

(4) $$\begin{array}{c} G_{ref}=\left\{\begin{array}{cc} & \frac{\left(m+1\right)A}{2\pi^{2}{D_{1}}^{2}{D_{2}}^{2}}\cdot \rho \cdot A_{wall}\cos^{m}\left(\phi \right)T\left(\theta \right)\cdot \\ & g\left(\theta \right)\cos\left(\theta \right)\cos\left(\alpha \right)\cos\left(\beta \right)\\ & \;\;\;\;\;\;\;\;\mathrm{if}\;\left|\frac{\theta }{FOV}\right|\leq 1,\\ & 0\\ & \;\;\;\;\;\;\;\;\mathrm{if}\;\left|\frac{\theta }{FOV}\right|>1 \end{array}\right. \end{array}$$

with the parameters being illustrated in Table 3.

Taking into consideration factors like background light intensity or other light sources, the above expression (3) can be expanded to

(5) $$\begin{array}{c} P_{r}=P_{rd}+P_{rnon-d}+P_{background} \end{array}$$

where $P_{background}$ is used to denote the additional light intensity. Interested readers are also referred to [49] and the references therein for further details of VLC channel models.

### 4.2. Positioning Techniques

There are various positioning algorithms proposed in the literature. A popular and practical solution is through triangulation, which is to determine the coordinates of a user (point) by knowing the distances (lateration) and/or angles (angulation) to/from the transmitters. The problem can thus be transformed to a traditional geometric problem. There are three kinds of algorithms based on triangulation: RSS, TDOA, and AOA methods. RSS and TDOA are lateration techniques, whereas AOA is an angulation technique. Please note that, apart from RSS-, TDOA-, and AOA-based methods, there exist alternative approaches based on proximity, fingerprinting, and image sensors. Additionally, hybrid systems have been developed to improve the overall precision. Further exploration and examination of various variations and alternative methodologies will be undertaken in subsequent discussions.

#### 4.2.1. RSS

Using the channel link gain models, the distances between the receiver and transmitters can be estimated from the received signal strengths. The lateration method can then be used to determine the exact position of the receiver.

In [50], a two-dimensional indoor localization system using RSS from LEDs was presented. The results of this study showed remarkable accuracy within centimeters for a room with dimensions of 6 m× 6 m and a height of 4 m. However, it is important to note that the system relies on certain assumptions, such as the receiver being positioned on the floor and both the receiver and transmitter axes being perpendicular to the ceiling where the LEDs are installed. Another related study [51] presents a system using four transmitters (LEDs). Each transmitter shares its coordinates through predefined time slots, creating a specific frame structure to maintain stable transmission power. By using RSS and solving a set of visible light transmission equations, the four channel link gains can be determined, facilitating the calculation of the receiver coordinates.

Different from [50,51], for interference avoidance among transmitters, the system in [52] considers a single transmitter but multiple (three) receivers. A receiver’s position can be estimated by determining the distances between the transmitter and receivers using RSS and by the given relative position of the receivers. The scheme in [53] extends the result of [52] by considering a single transmitter but using a single rotatable receiver. Due to the link gain difference according to the given azimuth angles, the user location can be estimated through the rotatable receiver.

In [54], a VLC-based IPS for an autonomous robot vehicle is proposed under the same assumption of [50]. By determining the relative ratio of signal strengths from each LED detected at the receiver according to the assigned individual time slot, circle or straight-line equations are obtained to derive their crossing point so as to indicate the user position. Note that the purpose of using the ratio of the received powers from each LED aims to cancel out the errors due to the difference in their optical device parameters.

In [55], a trilateration method is deployed to determine the receiver’s coordinates. Different frequency carriers are used at each transmitter for signal modulation, so the signals emitted from adjacent transmitters can be separated at the receiver without co-channel interference. Since the received power can vary due to the angle of radiation at the LED and the angle of incidence at the receiver, location estimation based solely on distance-dependent power attenuation is clearly inaccurate. By normalizing the radiation and incidence gains with optimized adjustment parameters, the experimental result shows that the average error of estimated positions can be reduced to a few centimeters.

In [56], a system consisting of four LED lamps, each composed of 16 LED chips (in a 4 × 4 array), is demonstrated. Each LED lamp is assigned a unique binary sequence (address vector) that is sent using VLC. The (x,y) coordinates of the receiver are then estimated by comparing the correlation value of the RSS and LED lamp addresses. The correlation value is defined as the inner product of the received signal and the address vector. If the position of the receiver is closer to a LED lamp, the correlation will be larger. Note that this is not sufficient to determine the exact position. For more accurate localization, a training phase similar to the Wi-Fi fingerprint method is provided to improve performance.

Unlike the systems mentioned above, the study presented in [57] considers the scenario where the receiver is not parallel to the ceiling but tilted, which is more realistic in practical use. As a result, many of the existing solutions are considered insufficient to provide high accuracy in situations involving user mobility or general cases. This paper provides a comprehensive analysis of the performance of the system with a tilted receiver. It also proposes the use of orientation information, e.g., from a gyroscope, to further improve the accuracy of the system.

It is important to note that a comprehensive theoretical accuracy evaluation of indoor positioning methods based on VLC using RSS is presented in [58], which also provides additional relevant references. Under typical parameter settings, the expected achievable accuracy can reach the centimeter level.

#### 4.2.2. TOA and TDOA

TOA is a distance estimation technique that measures the time it takes for a signal to travel from the transmitter to the receiver. Due to the high speed of light, achieving accurate distance measurements using TOA-based methods requires the use of highly accurate time measurement instruments. The research presented in [59] explores TOA-based techniques and establishes theoretical accuracy limits based on the Cramer–Rao Bound (CRB). It is important to note that TOA-based methods typically assume perfect synchronization between the transmitters and receiver for accurate time measurement, which can be challenging to achieve in practice. In comparison, time difference of arrival (TDOA)-based methods are generally preferred because they only require time synchronization between the transmitters.

Unlike the direct time measurement approach, the visible light indoor localization scheme proposed in [60], which is based on TDOA, measures the phase differences in the received signals from different transmitters (LEDs). In general, variations in the distance traveled by the light result in phase differences in the received signals. By achieving time synchronization among the transmitters, it becomes possible to calculate the distances between the LEDs and the receiver based on the observed phase differences. In [61], an enhancement to the aforementioned scheme is presented to tackle the impact of noise impairments. Considering that the received optical signal at the receiver is subject to additive white Gaussian noise (AWGN), error mitigation and reduction are achieved through the use of average filtering and maximum likelihood methods.

In [62], a 2D localization method based on estimating the TDOA from a pair of LEDs to a receiver is introduced. Instead of directly calculating the phase difference of the signals, the concept is to measure the peak-to-peak amplitude of the combined signals at the receiver. This is achieved by transmitting two sinusoidal signals of known frequencies from the LED pair. When the signals add constructively (in phase), the peak-to-peak amplitude reaches its maximum value, while destructive interference (out of phase) results in the minimum value. Using this technique, a trilateration-based localization scheme can be developed to determine the relative distances to the LEDs without having to explicitly calculate the phase differences between the received signals from different LEDs.

In [63], a low-complexity TDOA-based system is proposed for 3D localization using LEDs. Similar to [60], the distances between the LEDs and receiver are derived by measuring the phase differences between the received signals. Under the assumption that all the LEDs have the same height, a set of simultaneous linear equations can be constructed and solved to determine the coordinates of the receiver.

#### 4.2.3. AOA

Different from RSS- and TDOA-based methods, we can use the AOA to determine a receiver’s position. In [64], the receiver is a circular PD array composed of several PDs evenly distributed on a circle. The AOA from an LED to the receiver can be estimated according to the receiver’s orientation and the knowledge of the locations of the PDs on the circular PD array, so as to determine the coordinates of the receiver. Since the Lambertian received power equation can be regarded as a function of AOA, we can derive the AOA from the received power strength. It is also possible to estimate the distances between the LEDs and the receiver accordingly.

In [65], a visible light IPS is built based on a single transmitter and multiple tilted PDs. Inter-cell interference is negligible with a single transmitter. By measuring the received power strengths and thus the incidence angle gain differences due to different arrival angles and assuming that the tilted PDs are very close to each other so that they have the same coordinates, the receiver’s coordinates on a horizontal plane (2D) are first estimated. Then, by comparing the received power strengths against each possible vertical coordinate, the vertical distance (height) of the receiver is estimated to provide the 3D coordinates.

In [66], an optical LOS AOA-based positioning system is built. The mobile receiver contains an interior corner-cube formed by three orthogonal PDs to facilitate differential photo-current sensing and a retro-detection method [67] for estimating the azimuthal and polar angles of incident optical beams from LEDs to the receiver. Given the azimuthal and polar angles, a triangulation method is then applied to determine the 3D coordinates of the receiver. The experiment result with a sensitivity analysis of imbalanced signal strength to the positioning accuracy is provided and shows a much higher positioning accuracy than optical RSS-based positioning.

#### 4.2.4. Proximity-Based Methods

Proximity-based localization is simple but usually has low positioning accuracy. In VLC-based systems, LEDs are deployed as the reference beacons at known positions. A photodiode can detect whether the receiver module is located within the coverage range of a transmitting LED. For example, a proximity-based localization system using lighting LEDs is proposed in [68]. The identification data (ID) of LEDs are sent along with the illumination so that the receiver can identify the nearby LEDs (reference beacon positions) and thus estimate its approximate position.

In [69], a VLC proximity-based localization system is built using conventional LED lamps. Each lamp periodically broadcasts its coordinates and identification data. A photodiode is used to receive this information and determine its location in proximity to the lamps. By a mapping of the received LED lamp ID and geographic area, a simple indoor navigation application is demonstrated. A similar application of the above method for tracking visitors and providing navigation in museums is shown in [70]. A VLC receiver can also receive digital contents corresponding to his/her location in the museum.

To improve positioning accuracy, the VLC-based IPS in [71] embeds a six-axis geomagnetic sensor at the receiver for providing three axes of azimuth and three axes of tile angulation information for localization. Meanwhile, each LED light bulb broadcasts its coordinates as data through VLC technology. In addition, the impact of the maximum receivable signal incident angle (FOV) limit and optical receiver sensitivity limit to positioning accuracy is investigated. The experiment of an accurate IPS is demonstrated.

Another example of a proximity-based IPS is the angular diversity localization scheme used in [72]. The system contains a luminous LED array, with each LED transmitting at a different frequency, and has a lens located in front of the LED array so as to yield different light intersection regions due to different light overlapping patterns of the LEDs. Each region is distinguished by the presence or absence of different LED lights. The location of the receiver can thus be estimated by identifying each region. The above design does not require accurate light intensity or orientation measurements. Clearly, a larger number of LEDs in each luminary may create a larger number of distinguishable regions for possible smaller localization error. In general, the accuracy of the system will depend on the number of lights and also the receiver’s height.

#### 4.2.5. Fingerprint

RF-fingerprint-based positioning is a scene analysis method that compares the received information with a known information map to estimate the user position, which attracts much attention with the Wi-Fi technique. The principle is also applicable to a VLC-based system. The system in [73] is a two-dimensional IPS using fingerprinting of the power spectral density (PSD) of optical signals received from LEDs. During the offline initialization phase, prior knowledge of the PSD of each modulated visible light emitted by the LEDs is collected and stored as a database, namely a fingerprint map. For positioning, the real-time PSD received from a user can then be compared with that in the database for estimation in the online phase.

There are three main popular fingerprint-based indoor localization techniques [74], namely the probabilistic method, *k*-nearest-neighbor, and neural networks. In [75], a robust asynchronous indoor localization using modulated LEDs is built according to the Bayesian probabilistic estimation method, taking into account possible obstructions of the LoS from the beacons. Each LED broadcasts an embedded unique chip sequence as a beacon to allow mobile users to conduct localization. Given the chip sequences and the locations of the LEDs, a mobile device can synchronize and determine its position based on the likelihood of receiving each signal strength with respect to collected information during the offline training phase. Another example using the probabilistic method is [76], which estimates user location through best matching with a pre-recorded radio map of observed light intensities and signal patterns of modulated LED beacons that contains unique transmitter IDs.

In [77], the fingerprinting scheme estimates the position of a mobile device by comparing the received time samples of impulse responses to the collected power samples from the transmitters, known a priori, in the environment. In [78], the localization system exploits the signal extinction ratio distribution of LEDs. Each LED transmits its identification code in time division multiplexing in order to distinguish it.The receiver’s position is then estimated by comparing the received extinction ratio to the reference collected during the training phase.

#### 4.2.6. Image-Sensor-Based Schemes

Many systems use photodetectors or photodiodes as receivers. However, there are also systems that use image sensors or cameras. Algorithms using image sensors need to deal with the transformation of the two coordinate systems: the real world and the reception plane of the image sensor. The positions of the image sensor and LEDs should be known for setting up the transformation equations and their geometric relationships. The LEDs’ coordinates can be transmitted by broadcast through VLC and the reception plane coordinates can be derived from image processing. In [79], the algorithm proposes to use the co-linearity condition as well as least squares method to determine the transformation equations. Generally, the accuracy of the scheme can be improved when the number of pixels increases. Note that the cost of image-sensor-based schemes is usually higher due to the cost of image sensors. In addition, a compromise should be made between the accuracy and the running time of the image processing. In practice, one can also use a fish-eye camera [80] to have a larger field of view and to improve coverage.

In [81], the system contains two identical image sensors and four LED arrays while each pixel at the image sensors acts as an individual photo sensor. Similarly, the coordinate information of the LEDs is broadcast via VLC. According to the knowledge of the focal length of the image sensor lens, the locations of the image sensors, and the geometric relationship between the distance and the position difference of the LED images on the two image sensors, the coordinates of each image sensor can be calculated. Note that the algorithm does not require any angular measurement, such as AOA, but the separation of the image sensors has to be known with high accuracy for good performance.

#### 4.2.7. Hybrid Schemes

By combining multiple positioning techniques, such as RSS and AOA, the hybrid scheme can enhance the accuracy of the indoor positioning system. Moreover, the hybrid scheme provides redundancy and improves fault tolerance by using multiple positioning techniques. In [82], a VLC-based positioning system is built based on received light intensity and embedded accelerometer measurements, which are used to calculate the distances between the LEDs and the receiver. Accelerometers, on the other hand, are sensors that measure acceleration forces. The authors in [83] aim to enhance the accuracy and reliability of indoor positioning by incorporating accelerometers into the positioning system. The accelerometers can capture movement and orientation changes, which can be used to supplement the positioning information obtained from the visible light signals. By using the geometric relationship between the LEDs and receiver and the standard VLC channel model, the incidence of the receiver and the irradiance angles of the LEDs can be calculated so as to determine the position of the receiver [84].

The scheme in [41] is an enhanced system of [82] and very user-friendly for practical use. Note that the receiver is composed of at least three tilted PDs to acquire various measurements of different orientations from the LEDs instantaneously. According to experiments using four tilted PDs, the scheme is able to support high user mobility, with the receiver moving at the speed of 1.3 m/s, but still provides very reliable highly accurate user localization. The system proposed in [85] is a further refinement of the scheme in [41]. The system has only one PD on the user device to measure the light received through the projected area on the color-coded film. By comparing the measured light with a predefined color code, the user device can determine the AoA of the LED light and locate itself.

A hybrid algorithm is proposed by combining the utilization of RSS and AOA in [86]. If one technique fails or provides inaccurate results in a certain area, the system can rely on other techniques to ensure continuous and reliable positioning. However, it may be costly and complicated to implement and integrate these different technologies by adding additional hardware and software components.

#### 4.2.8. Hybrid Schemes

Before the end of this subsection, Table 4 shows a comparison of various VLP systems.

### 4.3. Multiple Access Schemes

Among the above positioning methods, different multiple access schemes have been deployed. In [60,61,62,63], frequency division multiplexing (FDM) is used by different pairs of LEDs such that the various TDOAs can be calculated simultaneously. In the AOA-based systems [73,90], FDM is also used for avoiding the inter-cell interference.

Among RSS-based systems and their variants, the positioning schemes in [41,51,54,82] use time division multiple access (TDMA), which would need very good time synchronization and a central unit to control all the transmitters. This often leads to a higher positioning accuracy of the system but at a high implementation cost. Note that a TDMA-based VLC system may often suffer from inter-pulse interference, which one can apply bit stuffing [92] to in order to migrate the interference between the light signals and to improve the illumination quality to allow a very short pulse width to be transmitted.

To avoid stringent time synchronization, code division multiple access can be used to distinguish signals from different transmitters. In [93], each transmitter is assigned a unique optical orthogonal code (OOC). Signals from different transmitters can be separated at the receiver according to their correlation properties. In [94], a novel multiple access scheme for decentralized asynchronous VLC systems is proposed considering that the transmitters and receivers cannot be synchronized. Notice that there is often a trade-off between the number of available codes and their sequence length. To support a large number of transmitters, one can consider a spatial reuse method when there is a limited number of the codes for a large area with many transmitters. A fully distributed code division multiple access scheme is proposed in [95] that only requires chip-level synchronization. The simulation result shows that the average BER performance of the scheme is worse than TDMA but much better than FDMA.

Even in the scenarios where time synchronization can be achieved, TDMA has been shown to be suboptimal [96]. Pilot sequence designs are proposed in [96] for simultaneously estimating the channel gains of multiple transmitters in a lighting system and satisfying the average and maximum transmit power constraint due to the illumination purpose. The paper illustrates how to design the optimal pilot sequences that minimize the noise variance experienced by a receiver, although the optimization problem is non-convex. For systems with ambient light and systems without ambient light, it also reveals the fundamental trade-off between noise variance, average transmit power, maximum transmit power, number of light sources, and pilot length.

The system in [97] uses a dual-tone multi-frequency (DTMF) technique for multiplexing such that each LED will transmit using a unique ID of several digits that is represented by a combination of low and high frequencies. The advantage of DTMF is that the system can easily separate different received signals by analysis in their frequency and time domain.

Another solution is framed slotted ALOHA (FSA) in [50]. Due to asynchronous random multi-access, transmission collisions are expected. So, this system would require a large number of slots in a frame to maintain a high probability of successful transmission for a given number of users. For example, in [98], the authors use a frame structure containing 400 slots in each frame to provide transmission reliability.

## 5. Algorithms for VLC-Based IPS

**Figure 3:**
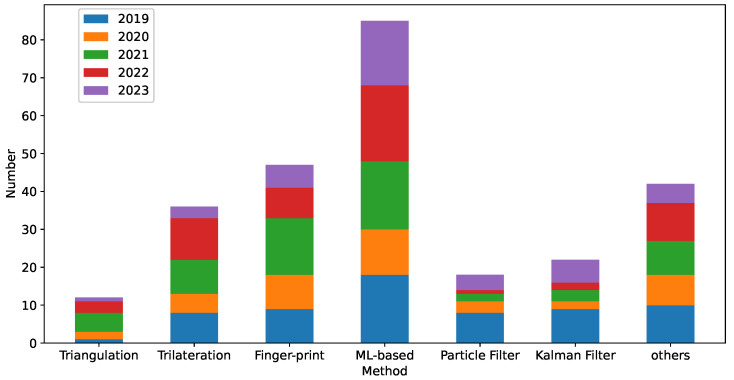
The number of papers for different algorithms.

By leveraging the existing techniques, VLC-based IPSs offer a cost-effective and energy-efficient approach to accurately locate and track objects or individuals within indoor environments. However, the development of efficient algorithms for VLC-based IPSs faces various challenges. The complexity of such algorithms depends on the intricacies of channel modeling, signal processing, and positioning techniques. Channel modeling involves understanding the behavior of light propagation in indoor environments, which can be affected by factors such as reflection, diffraction, and interference. Signal processing techniques are crucial for extracting useful information from the received light signals, considering the noise and interference present in the environment. Several approaches can be proposed to optimize VLC-based IPSs. This section provides a comprehensive review of the extensively researched algorithms. We have collected relevant research papers from Google Scholar in recent years, and the statistical data are shown in the above Figure 3.

### 5.1. Trilateration and Triangulation Algorithms

#### 5.1.1. Trilateration Algorithms

**Figure 4:**
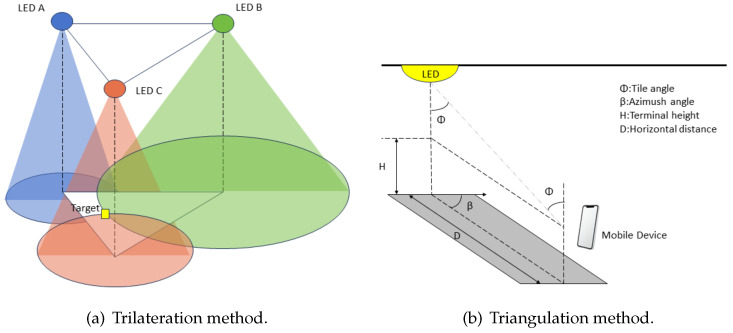
(**a**) Trilateration [101] and (**b**) triangulation [102] algorithms.

To implement trilateration algorithms, at least three visible light sources are required for three-dimensional (3D) localization [99]. These light sources emit light signals, and the receiver measures the signal strength or time-of-flight (ToF) of these signals. A typical scheme is shown in Figure 4a. Once the distances between transmitters and receivers are determined, one can construct circles centered at each light source with radii equal to the distances. The receiver’s position is then estimated as the intersection point of these circles. The intersection point represents the receiver’s location in the environment.

It is worth noting that the accuracy of the trilateration algorithm depends on the accuracy of distance measurements and the geometry of the visible light sources. Factors such as signal attenuation, interference, and multipath effects can affect the accuracy of the localization. In [100], the coordinates or orientation of a receiver, which can have either multiple or single photodiodes, are determined using the information about spherical coordinates that represent the orientation of LEDs.

The trilateral measurement algorithm is the basis for the VLC-based indoor positioning system and provides a reliable method for determining a user’s exact position. However, the effectiveness of the algorithm would much rely on the accurate distance measurements and the ability to mitigate errors caused by environmental factors.

#### 5.1.2. Triangulation Algorithms

Similar to trilateration-based algorithms, triangulation also requires multiple visible light sources with known positions for 3D localization as shown in Figure 4b. We can create the triangle for locating the mobile device by measuring the angles between the receiver and at least three light sources. The known positions of the light sources act as the vertices of the triangle, and the position of the receiver is determined by finding the intersection point of the lines or angles formed by the receiver and the light sources. The authors in [103] propose the indoor robot positioning algorithm based on triangulation using a multi-frequency approach. In [104], a triangulation-based method is proposed to estimate the receiver position using RSS with a minimum of three distance estimates.

The triangulation algorithm is commonly used in VLC-based IPSs since it can provide accurate location estimation. However, it often requires precise angle measurements and knowledge of the light source positions, which may be challenging in real-world environments due to factors like noise, interference, and multipath effects.

### 5.2. Fingerprinting-Based Algorithms

**Figure 5:**
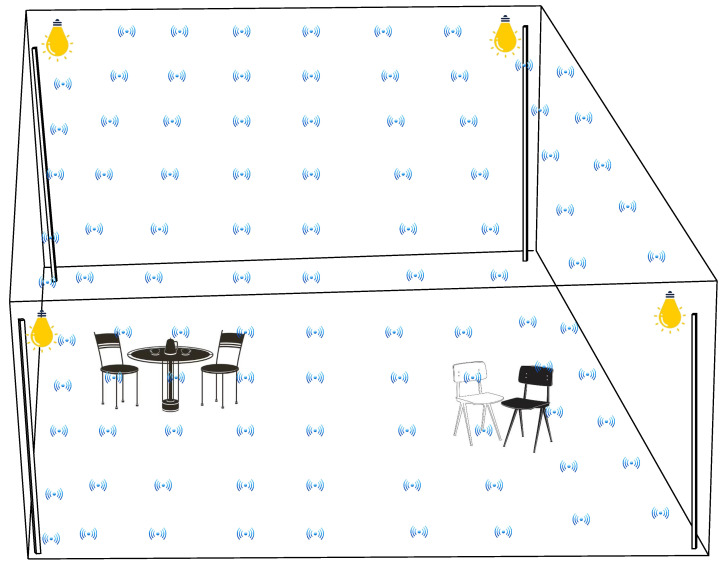
Indoor positioning based on the three-dimensional signal fingerprints collected at various locations [107].

In contrast to the trilateration and triangulation algorithms, fingerprinting-based algorithms as illustrated in Figure 5 rely on constructing a database of signal fingerprints at known locations. During the positioning phase, the algorithm compares the received signal fingerprints with the database to estimate the position of the receiver.

The offline phase involves collecting signal fingerprints at various known locations within the indoor environment [18]. This typically requires surveying the environment and measuring the RSS or other relevant signal parameters at each location. These measurements are then stored in a database along with their corresponding known positions. During the online phase, when the receiver position needs to be estimated, the algorithm compares the received signal fingerprints with the pre-trained fingerprints in the database [105]. By finding the closest match or applying probabilistic techniques, the algorithm can estimate the receiver’s position based on the similarity between the received signal and the stored fingerprints. In [106], an accurate indoor VLC-based positioning system is implemented using a modified path loss model with space fingerprints. This approach is specifically designed to meet the requirements of site surveying, achieving accurate positioning even in NLOS scenarios with limited offline measurements.

Fingerprinting-based algorithms have the advantage of being able to operate without prior knowledge of the signal propagation model for the environment, as long as the database has been prepared in the offline phase. However, they can be sensitive to changes in the environment, such as variations in signal propagation due to obstacles or interference. Regular updates to the fingerprint database may be required to account for such changes [106].

### 5.3. Machine-Learning-Based Algorithms

To cope with the vulnerabilities of fingerprinting-based algorithm, ref. [108] presents several machine-learning-based algorithms, which leverage the power of statistical models to learn patterns and relationships from labeled training data. Support vector machines (SVMs), k-nearest neighbors (KNNs), and deep learning (DL) are some of the popular machine learning algorithms used in indoor positioning. The authors in [109] explore the SVM-based algorithm by using variations in the RSS to increase the accuracy and reduce the computation complexity. KNN is a simple algorithm that classifies an object based on its nearest neighbors; see, for example, [106]. In addition, deep-learning-based algorithms have been widely explored in recent years. For instance, refs. [110,111,112] propose several DL-based algorithms with diverse sensors.

These algorithms require substantial amounts of data to learn patterns and make accurate predictions. Acquiring and labeling such data can be time-consuming and costly. Moreover, machine learning algorithms may struggle to generalize well to unseen data if the training data are not representative of the real-world scenarios that they will encounter.

Machine-learning-based algorithms can overcome several limitations of traditional methods. They can handle possible complex nonlinear properties among various system variables that are often difficult to be handled by traditional methods due to the complexity. In addition, these algorithms can have higher anti-noise ability, e.g., one can reduce the impact of noise on localization performance by training the model on data containing noise. In addition, deep learning algorithms can be used to automatically extract useful features for localization, reducing the effort of manual feature engineering, and discover hidden features that cannot be easily captured by traditional methods. It is also worth noting that machine learning algorithms are often able to adapt to dynamic environments by constantly updating and retraining models, making positioning systems more flexible and effective in practical applications.

### 5.4. Filtering-Based Approaches

Particle filtering (PF) and Kalman filtering (KF) are two commonly used probabilistic estimation methods in IPSs. PF is a non-parametric filtering technique [113]. It represents the possible receiver positions using a set of particles and assigns weights to each particle based on the likelihood of the received signal measurements. The weights are then updated using a re-sampling step, where particles with higher weights are more likely to be selected for the next iteration. This allows the particle filter to track the receiver’s position over time and provides an estimate of its location. On the other hand, KF is a recursive estimation algorithm that combines a prediction model and a measurement model. It assumes that the system being tracked follows a linear dynamical model with Gaussian noise. KF iteratively updates the estimate of the receiver’s position based on the prediction from the previous state and the new measurement [114]. The filter also estimates the uncertainty or covariance of the position estimate, which provides a measure of the confidence in the estimate.

Both PF and KF have their own advantages and limitations [115]. PF can handle non-linear and non-Gaussian systems, but it requires a large number of particles to achieve accurate results, which can be computationally expensive. On the other hand, KF is computationally efficient and provides optimal estimates for linear and Gaussian systems, but it may struggle with non-linear or non-Gaussian systems.

In practice, a combination of these two methods, known as PF-KF, can be used to improve the accuracy and efficiency of indoor positioning systems [116]. PF is used to handle the non-linear and non-Gaussian aspects of the system, while KF is used to refine the estimates and reduce the computational complexity.

## 6. Performance Evaluation and Benchmarking

### 6.1. Metrics for Evaluating VLC-Based IPS

#### 6.1.1. Accuracy

Euclidean distance is a straightforward metric that measures the straight-line distance between the estimated and true position. The formulation can be represented as follows:

(6) $$d=\sqrt{{\left(p_{est}-p_{true}\right)}^{2}}$$

where $p_{est}$ and $p_{true}$ are the estimated and true positions, respectively.

#### 6.1.2. Precision

Precision measures the consistency of the estimated positions. It indicates how closely the estimated positions are clustered together. MSE is a commonly metric that measures the average squared difference between the estimated and true positions. The metric can be formulated as

(7) $$\epsilon =\frac{1}{N}\sum_{i}^{N}{\left(p_{est,i}-p_{true,i}\right)}^{2}$$

where *N* is the total number of samples and the subscript *i* represents the *i*-th measurement.

#### 6.1.3. Robustness

Robustness can be quantified by identifying and counting the number of outliers in the localization results. Outliers are data points that significantly deviate from the expected value and can negatively impact the accuracy of the system. Various statistical techniques, such as clustering, filtering, or robust regression, can be employed to detect and mitigate the effect of outliers.

For instance, the authors in [117] propose a two-phase indoor positioning algorithmic framework, namely *coarse phase* and *fine phase*, when subject to insufficient anchor luminaries. In [118], a continuously adaptive meanshift algorithm is performed to improve the robustness. Even if most of the LED is shielded or damaged, the system can still achieve high-precision tracking by utilizing color recognition and local detection, regardless of changes in shape. This ensures robust positioning capabilities without affecting the real-time ability of the positioning algorithm.

#### 6.1.4. Computational Complexity

Computational complexity is one of the most important aspects to consider. In general, the complexity of a VLC-based IPS is due to the channel modeling between transmitters and receivers [119]. Factors such as reflections, diffraction, and interference need to be taken into account [120]. In addition, the complexity of signal processing algorithms used for demodulation, decoding, and localization will contribute to the overall complexity of the system. Furthermore, advanced techniques like multi-user detection and channel estimation can improve localization accuracy but would also lead to higher computational complexity. Thus, the trade-off between accuracy and complexity should be carefully considered.

#### 6.1.5. Hardware Cost

As compared to conventional approaches in wireless technologies such as Wi-Fi and Bluetooth, VLC-based IPSs have several advantages, including high accuracy, low latency, and immunity to electromagnetic interference. However, one of the critical factors that can limit the wide adoption of VLC-based IPSs is the hardware cost. Deploying a high density of light transmitters in an indoor environment may become expensive, making it challenging to scale these systems to large deployments. Therefore, hardware cost is an important metric when evaluating the feasibility and commercial viability of VLC-based IPS solutions.

Hardware costs can vary significantly depending on the components and design choices made during the system development phase. For instance, the cost per node can increase if high-resolution cameras or complex signal processing algorithms are used. Additionally, designing customized hardware components can also add to the total cost of the system. Therefore, it is essential to consider the hardware cost metric while designing and developing VLC-based IPSs, particularly when aiming for large-scale deployment.

### 6.2. Research Challenges and Future Work

Despite the multitude of proposed solutions for VLC-based IPSs, there are still several areas that require additional attention and further research.

#### 6.2.1. Noise and Interference

Many systems and their designs do not manage noise and interference or over-simplify the modeling. For example, in [48,121], the modeling of the channel multipath only assumes light power due to the first reflection. However, can we simply ignore the light power arriving at a transmitter after several reflections, for example, due to the walls and environment? In addition, in many experiments and simulations, results consider only direct light and ignore the impact of multipath signal strength [54,56,60,73]. Sometimes, it is even assumed that the environment is noiseless or that the experiment is conducted in the dark. Some also consider that the interference due to other light sources can be avoided due to the use of different frequencies [14]. However, it should be noted that ambient light cannot be ignored for the accuracy of positioning.

In general, the noise in VLC measurement [48,51,121] can be expressed as

(8) $$\begin{array}{c} \sigma_{noise}^{2}=\sigma_{thermal}^{2}+\sigma_{shot}^{2} \end{array}$$

where $\sigma_{thermal}^{2}$ denotes the thermal noise in the optical channel while $\sigma_{shot}^{2}$ denotes the shot noise, which depends on the light-induced current and can be modeled as Gaussian noise.

There are some solutions to measure the noise level in the visible light channel. In [41,82], the first time slot in the TDMA is reserved for measuring the background power while all the transmitters are turned off. As shown in [96], coding can be used to minimize the noise variance experienced by receivers in a system where background power is estimated. On the other hand, the scheme in [92] uses the last few bits to measure the ambient noise, which is similar to the solution in [41,82].

In [122], the authors investigate the impact of a multipath in VLC. The results show that the impact is more severe when the receiver device is located at the corner or the edge of a room environment compared to that when the receiver device is located in the center of the room [77,93]. It is also shown that the multipath can cause a more significant impact to indoor positioning than that due to shot noise or thermal noise, so the multipath cannot be ignored. Until now, there have not been many research studies focused on how to mitigate this kind of influence.

#### 6.2.2. Ease of Use and Robustness

VLP systems have significant potential for widespread adoption in everyday devices if they can be easily implemented and designed to be user-friendly. However, many existing approaches have only been demonstrated in specific scenarios. For example, the positioning accuracy of the system in [73] was primarily tested in dark environments. Furthermore, experimental results and performance evaluations are often conducted in simplistic laboratory settings, such as unobstructed environments, which do not accurately reflect the complexity of living spaces. It is also important to note that some systems require a preliminary training phase prior to deployment, requiring additional preparation time and effort [73,79]. This training phase can be particularly resource-intensive when adapting to new or modified environments.

Evaluating performance in different work environments is critical to ensuring the robustness of positioning systems [14]. Examples of such environments include large conference rooms, small cubicles, and conventional corridors. It is important to recognize that variations in the number of available transmitters can significantly affect the positioning accuracy and continuity of service. Furthermore, accommodating user mobility is essential for the practical application of these systems [41]. In this context, the design of the receiver plays a key role in providing highly accurate positioning information to user applications.

#### 6.2.3. Hybrid System

Future applications will need to support positioning in both indoor and outdoor environments, ideally using the same user terminal, such as a mobile device. GPS services are already widely used for outdoor positioning, while VLP (visible light positioning) systems are well suited for indoor services. Hybrid systems that combine VLC-based technologies with WiFi, Bluetooth, cellular networks, and others to provide seamless and reliable location services in all environments would also be very practical. The advantage of hybrid systems is that they allow for a seamless transition between indoor and outdoor environments. For example, users can use GPS to navigate outdoors, and, when they enter a building, the system automatically switches to VLP or WiFi positioning, ensuring that users always have access to highly accurate location services.

In addition, by combining different positioning technologies, the hybrid system can significantly improve positioning accuracy and robustness and adapt to complex environmental changes and different usage scenarios, providing a better user experience and a wider range of applications. In this way, the hybrid system not only improves the reliability and accuracy of location services but also provides a solid foundation for future smart cities, intelligent transportation, and other IoT applications.

## 7. Conclusions

This paper presents a comprehensive overview of visible light communication (VLC)-based indoor positioning systems (IPSs), comparing and analyzing different positioning technologies. VLC-based systems differ from traditional solutions by offering unique features and novel advantages. This study discusses their system designs, including channel modeling, localization algorithms, and multiple access schemes, and provides practical implementation and usage guidelines.

Key findings and comparisons are succinctly summarized in Table 4, facilitating a deeper understanding of the strengths and weaknesses inherent in different VLC-based IPSs. In addition, this paper identifies future research directions and challenges that need to be addressed to further improve the performance and applicability of VLC-based positioning systems. Although VLC-based IPSs perform well in many ways, factors such as multipath effects, signal occlusion, and ambient light interference can affect positioning accuracy. In addition, there are some practical issues that need to be addressed when deploying and maintaining the system, such as the optimal layout of the LED and the sensitivity adjustment of the receiver. Overall, this paper contributes to the advancement of indoor positioning technologies by highlighting the potential of VLC-based systems and providing valuable insights for researchers and practitioners in the field. With continued research and development, VLC-based IPSs are poised to revolutionize location-based services by significantly improving the accuracy and reducing the cost of indoor positioning.

## Figures and Tables

**Table 1:** Comparison of different positioning systems.

Technology	Accuracy (m)	Cost
GPS	5–40	High
Cellular	5–50	High
Wi-Fi	1–5	Low
RFID	1–2	Low
Infrared	1–2	Medium
Bluetooth	1–5	Low
Ultrasonic	0.03–1	High
UWB	0.1–1	High
VLC	0.05–0.1	Low

**Table 2:** The parameters for direct light channel model.

Parameter	Explication
*m*	Lambertian parameter with $m=-\log2/\log\left(\cos\phi_{\frac{1}{2}}\right)$
ϕ	Angle of irradiance
$\phi_{\frac{1}{2}}$	Half-power angle of LED
θ	Angle of incidence
FOV	Angle of a field of view at the receiver
A	Effective area of the photodiode
$D_{d}$	Direct distance between transmitter and receiver
$T\left(\theta \right)$	Filter gain
$g\left(\theta \right)$	Concentrator gain

**Table 3:** The parameters for reflected light channel model.

Parameter	Explication
$P_{rnon-d}$	Sum of received reflected power
$D_{1}$	Distance between the LED and a reflective point
$D_{2}$	Distance between the reflective point and the receiver
ρ	Reflectance factor
$A_{wall}$	A reflective area of small region
ϕ	Angle of irradiance to a reflective point
α	Angle of incidence to a reflective point
β	Angle of irradiance to the receiver
θ	Angle of incidence

**Table 4:** Comparison of different VLC-based indoor positioning systems. S and P refer to simulation and practical experiment, respectively.

	Receiver	Algorithm	Complexity (Evaluation Method)	Multiple Access Scheme	Operating Range (m)	Accuracy (cm)	Assumptions
[54]	PD	RSSR	Simple (P)	TDMA	1 × 1 × 1.3	Max 10.29, mean 3.24	Distortionless, only directed light, LEDs and receiver are parallel
[55]	PD	RSS	Simple (P)	FDMA	equilateral triangle of 0.6 height 0.6	2.4 with adjustment process	Distortionless, transmitters should be fixed at the same height
[51]	PD	RSS	Simple (S)	TDMA	3 × 3 × 3	<0.05	Distortionless, only directed light, LEDs and receiver are parallel
[14]	PD + IMU	RSS	Simple (P)	Random channel hopping	Conference room, cubicle area, corridor	Medium 30	LEDs and receiver are parallel
[87]	PD	RSS correlation	Simple, needs training stage (S)	CDMA	5 × 5 × 6	<10	Optical channels are line-of-sight (LOS) links
[52]	Multiple PDs	RSS	Complex (S)	No need	2 × 2 × 2	<1.5	There is only one transmitter, LEDs and receiver are parallel
[88]	PD	AOA	Complex (S)	FDM	5 × 5 × 3	Max 1.4	Distortionless, only directed light
[61]	PD	TDOA	Complex (S)	FDM	5 × 5 × 3	<2	The noise of system is considered as AWGN
[89]	PD	TDOA	Complex (S)	TDMA	5 × 5 × 3	Mean 3.9	The transmitter IDs of received signals are unknown. But, the number of transmitters is known
[63]	PD with extra equipment	TDOA	Complex (S)	FDM	5 × 5 × 3	Mean 0.02	Distortionless, only directed light, transmitters should be fixed at the same height
[65]	Tilted multiple PDs	AOA+RSS	Complex, need training stage (S)	No need	2 × 2 × 2.5	<6	Azimuth angles and polar angles are fixed and known
[90]	Differential photo sensor	AOA	Complex (S)	FDM	Not mentioned	RMS 3.7	Photo sensor is fixed and position of optical beacon changes
[82]	PD + IMU	AOA	Complex (S)	TDMA	5 × 3 × 3	<25	Transmitters should be fixed at the same height
[41]	PDs + IMU	AOA	Complex (P)	TDMA	5 × 4 × 3	<6 (at 1.3 m/s)	No special assumption
[91]	PD	RSS	Simple (P)	FDMA	0.75 × 0.75 × 2	Positioning error in the area of 1 m × 1 m × 2 m can reach within a few centimeters	Less light interference and reflection
[75]	PD	Fingerprinting	Complex (S)	CDMA	30 × 30 × 30	RMS 81	There is the possibility of obstructions
[77]	PD	Fingerprinting	Complex (S)	Not mentioned	10 × 9 × 3.1	max 80	The reflectivity of obstructions is taken into account but not other noise
[78]	PD	Fingerprinting	Simple (S)	TDM	0.9 × 0.9 × 1.5	Mean 1.5787	The positions in simulation limits in a 60 cm equilateral triangle
[73]	Multiple PDs	Fingerprinting	Complex, need training stage (P)	FDM	1.8 × 1.2 × 1	Mean 14.8674	Experiment tested in dark
[79]	Image sensor	Co-linearity + image processing	Complex (S)	No	Street	<150 cm (pixels)	The received point and the center of the lens of receiver must be on the same straight line

## Abbreviations

The following abbreviations are used in this manuscript:

AOA	Angle of arrival
AP	Access point
GPS	Global positioning system
IoT	Internet of Things
IPS	Indoor positioning system
LEDs	Light-emitting diodes
LOS	Line-of-sight
NLOS	Non-line-of-sight
RFID	Radio-frequency identification
RSS	Received signal strength
TOA	Time of arrival
VLC	Visible light communication
VLP	Visible-light-communication-based positioning
WLAN	Wireless local area network
